# Cellular Interactions in the Intestinal Stem Cell Niche

**DOI:** 10.1007/s00005-018-0524-8

**Published:** 2018-09-21

**Authors:** Agnieszka Pastuła, Janusz Marcinkiewicz

**Affiliations:** 1 Clinic and Polyclinic for Internal Medicine II, Klinikum Rechts der Isar, Technical University of Munich, Munich, Germany; 2 Chair of Immunology, Jagiellonian University Medical College, Czysta 18, 31-121 Kraków, Poland; 3 Department of Genetics and Development, Columbia University Medical Center, New York, NY 10032 USA; 4 Department of Medicine, Columbia University Medical Center, New York, NY 10032 USA; 5 Department of Urology, Columbia University Medical Center, New York, NY 10032 USA; 6 Herbert Irving Comprehensive Cancer Center, Columbia University Medical Center, New York, NY 10032 USA

**Keywords:** Mesenchymal–epithelial cross-talk, Inflammatory bowel disease, Myofibroblasts, Intestinal stem cells, Stem cell niche

## Abstract

Epithelial cells are one of the most actively cycling cells in a mammalian organism and therefore are prone to malignant transformation. Already during organogenesis, the connective tissue (mesenchyme) provides instructive signals for the epithelium. In an adult organism, the mesenchyme is believed to provide crucial regulatory signals for the maintenance and regeneration of epithelial cells. Here, we discuss the role of intestinal myofibroblasts, α-smooth muscle actin-positive stromal (mesenchymal) cells, as an important regulatory part of the intestinal stem cell niche. Better understanding of the cross-talk between myofibroblasts and the epithelium in the intestine has implications for advances in regenerative medicine, and improved therapeutic strategies for inflammatory bowel disease, intestinal fibrosis and colorectal cancer.

## Introduction: Tissue Microenvironment

Epithelial stem cells are responsible for the normal epithelial tissue regeneration in an adult organism for example in skin, respiratory tract and gastrointestinal tract. Epithelial stem cells are constantly interacting with the local surroundings, known as the stem cell niche, which is composed of extracellular matrix (ECM), soluble factors and mesenchymal cells. Among mesenchymal cells, we can distinguish, for example, different types of immune cells, endothelial cells, neurons, mesenchymal stem cells, fibroblasts and myofibroblasts. In this review, we focused on the role of mesenchymal cells, particularly intestinal myofibroblasts (IMFs), as a crucial component of the intestinal stem cell niche.

### The Intestinal Stem Cell Niche

Intestinal epithelial cells are one of the most actively cycling cells in our body, and they are also prone to malignant transformation. High cell turnover in the intestinal epithelium is fueled by the intestinal stem cells (ISCs) that are located at the bottom of the intestinal crypt. ISCs can be distinguished from other cells by the expression of Lgr5 (Barker et al. 2007), which marks actively cycling ISCs. In addition, it is believed that there exists another subpopulation of ISCs that are quiescent and are marked with Bmi1 (Sangiorgi and Capecchi 2008), or other markers such as: Hopx, mTERT and Lrig1 (Barker et al. 2012). The intestinal epithelium is heterogenous as it is composed of different epithelial cell types such as enterocytes, enteroendocrine cells, goblet cells and Paneth cells. A source for all those epithelial cell types is an ISC. Recently, Paneth cells have been shown to be a crucial component of the intestinal stem cell niche and provide niche factors for ISCs (Sato et al. 2011). However, depletion of Paneth cells did not cause significant alterations in the intestinal crypt (Durand et al. 2012) thus suggesting that mesenchymal cells provide essential niche factors for the ISCs.

### Mesenchymal–Epithelial Cross-Talk in the Intestinal Stem Cell Niche

Many studies have provided evidence on the importance of the mesenchymal–epithelial cross-talk in the intestinal stem cell niche (Table 1). First, Foxl1^+^ mesenchymal cells were shown to regulate proliferation in the intestinal crypt (Aoki et al. 2016). Second, Wnt5a^+^ mesenchymal cells were demonstrated to stimulate epithelial regeneration in an acute intestinal damage model (Miyoshi et al. 2012). Third, a study of Miyoshi et al. suggests that mesenchymal cells can also play an important role during intestinal epithelial recovery after chemotherapy-induced damage (Seiler et al. 2015). In addition, interference with the bone morphogenetic protein signaling in the stroma has a profound impact on the epithelium as it results in the growth of polyps (Beppu et al. 2008). Importantly, mesenchymal–epithelial cross-talk is not unidirectional, also the intestinal epithelium provides signals to the adjacent stroma as it was demonstrated by Madison et al. (2005). In this study, the authors showed that reduction of Sonic (Shh) and Indian (Ihh) hedgehog, that are expressed in the intestinal epithelium, results in mislocalization of subepithelial myofibroblasts (Madison et al. 2005).

**Table 1:** Examples of the mesenchymal–epithelial cross-talk in the intestine

Description	References
Deletion of the BMP type II receptor in the stroma induces formation of intestinal polyps	Beppu et al. (2008)
Intestinal epithelium provides hedgehog signals to subepithelial myofibroblasts and smooth muscle cells	Madison et al. (2005)
Deletion of Foxl1^+^ mesenchymal cells reduces epithelial cell proliferation in the intestinal stem cell niche. Moreover, Foxl1^+^ mesenchymal cells are a source of Wnt ligands in the intestinal stem cell niche	Aoki et al. (2016)
Subepithelial cells are involved in regeneration of the intestinal epithelium after doxorubicin-induced damage	Seiler et al. (2015)
Wnt5a^+^ mesenchymal cells are involved in the repair of the intestinal epithelium in biopsy-injured mice	Miyoshi et al. (2012)

*BMP* bone morphogenetic protein

In the intestinal stem cell niche, there are phenotypically and functionally distinct populations of mesenchymal cells such as: alpha-smooth muscle actin (α-SMA)^+^ myofibroblasts (Powell et al. 1999b) and α-SMA^−^ mesenchymal cells, e.g., CD34^+^ mesenchymal cells (Stzepourginski et al. 2017) and Foxl1^+^ mesenchymal cells (Aoki et al. 2016). Here, we focused on the α-SMA^+^ myofibroblasts, because they are present not only in an adult organism, but also during early intestinal development (Artells et al. 2011). This suggests that α-SMA^+^ IMFs could: (1) regulate intestinal morphogenesis; (2) provide key niche signals for proliferation and differentiation of both fetal and adult intestinal epithelium. Moreover, α-SMA^+^ myofibroblasts have important implications for cancer research.

## Myofibroblasts

### Multiple Functions of Myofibroblasts

Myofibroblast is a spindle-like, contractile cell that has a mesodermal origin and expresses α-SMA. Myofibroblasts are responsible for the production of ECM proteins (Frantz et al. 2010), which provide a scaffold for the tissue and growth factor signaling. Besides that, myofibroblasts secrete a broad spectrum of growth factors, proteases, cytokines, and chemokines (Powell et al. 1999a). Myofibroblasts are involved in many processes in a mammalian organism. Myofibroblasts play an important role during development (Mitchell 2005), angiogenesis (Mayrand et al. 2012) and immune response (Andoh et al. 2007; Otte et al. 2003). Moreover, myofibroblasts are critical players during wound healing, where they are responsible for contractility of an injured area and formation of a scar (Gabbiani 2003; Klingberg et al. 2013). Myofibroblasts are implicated in many diseases such as liver cirrhosis, renal fibrosis or lung fibrosis (Gabbiani 2003; Klingberg et al. 2013; Meran and Steadman 2011), and cancer. At the tumor niche, myofibroblasts are one of the most abundant non-malignant cell type and promote tumor progression (Cirri and Chiarugi 2012; Orimo and Weinberg 2006; Quante et al. 2011). Myofibroblasts are recognized as potential targets for both fibrotic diseases (Scotton and Chambers 2007) and cancer (Micke and Ostman 2004). Moreover, IMFs along with crypt epithelial cells express Toll-like receptors that points to their ability to cross-talk with gut microbiota products and their impact on mucosal immunity (Brown et al. 2014).

### Subepithelial Myofibroblasts in the Intestine

In the intestine, those myofibroblasts that are adjacent to the intestinal epithelium are known as subepithelial myofibroblasts or pericryptal myofibroblasts. The intestinal crypt is composed of about 250 epithelial cells, including 15 Lgr5^+^ stem cells (Clevers 2013). Each day about 200 new crypts are generated. About 38 myofibroblasts in the small intestine and 124 myofibroblasts in colon form a niche around a crypt (Neal and Potten 1981). Those myofibroblasts are α-SMA^+^, vimentin^+^ and desmin^−^ cells, and are slowly cycling, and fuse with each other to form syncytia (Powell et al. 1999b). A recent study of Sacchetti et al. (2017) suggests that expression of microRNA-204&211 can distinguish subepithelial myofibroblasts from α-SMA^−^ mesenchymal stromal cells. Nevertheless, both microRNAs as well as well-known mesenchymal cell markers, e.g., α-SMA, vimentin and desmin, exhibit intracellular localization. Hence, there is an urgent need to identify novel stromal cell markers that belong to the group of cell surface proteins, so that they could be used for fluorescence-activated cell sorting (FACS) of the mouse as well as human tissue that will certainly accelerate progress in understating the contribution of stromal cells to chronic diseases of the gastrointestinal tract.

**Fig. 1:**
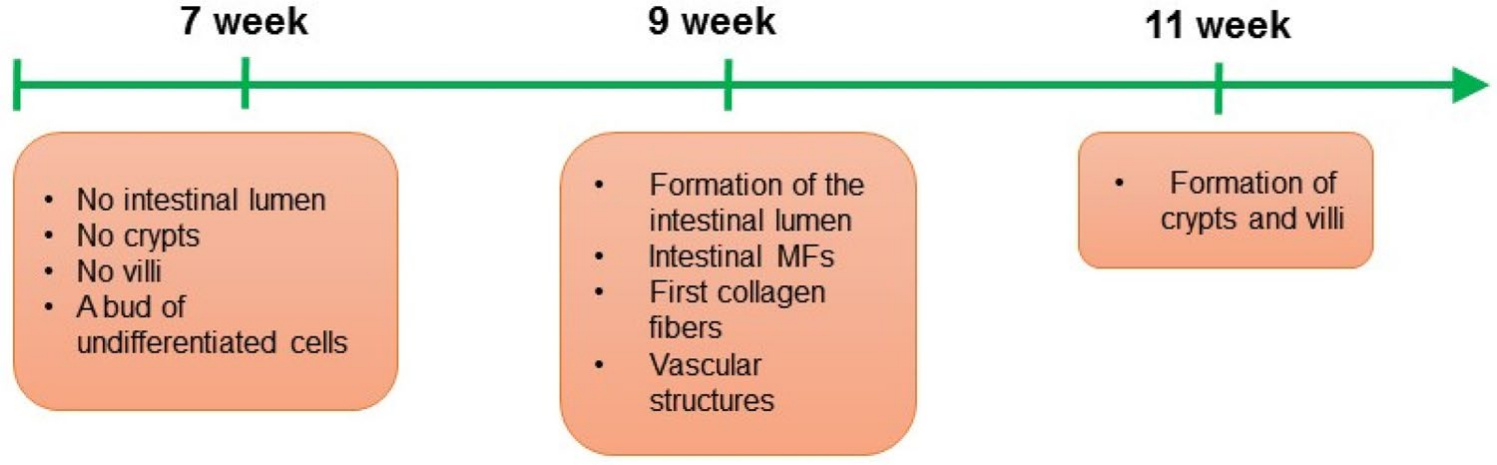
Organogenesis of human small intestine and initiation of the myofibroblast (MF)–epithelium interactions in the intestinal stem cell niche. During the 7 weeks of small intestine human development, a bud of undifferentiated cells is observed, at that time point crypts and villi are not formed yet. During the 9 weeks of small intestine human development, the intestinal lumen is initiated, and the first intestinal MFs, vascular structures and collagen fibers are detected. During the 9 weeks of small intestine human development crypts and villi are present

Transplantation studies demonstrated that subepithelial myofibroblasts in the intestine in both mice and human originate from bone marrow (Brittan et al. 2002). Besides that, myofibroblasts can originate from local fibroblasts and local mesenchymal stem cells, gremlin^+^ intestinal reticular stem cells, fibrocytes, and as result of the epithelial–mesenchymal transition (EMT) (Artells et al. 2011; Micallef et al. 2012; Worthley et al. 2015). IMFs appear for the first time during the 9 weeks of human development (Artells et al. 2011). Excitingly, appearance of myofibroblasts correlates with formation of the intestinal lumen (Artells et al. 2011) (Fig. 1), which implies that this stromal cell type can play a crucial role during the intestinal epithelial morphogenesis.

**Fig. 2:**
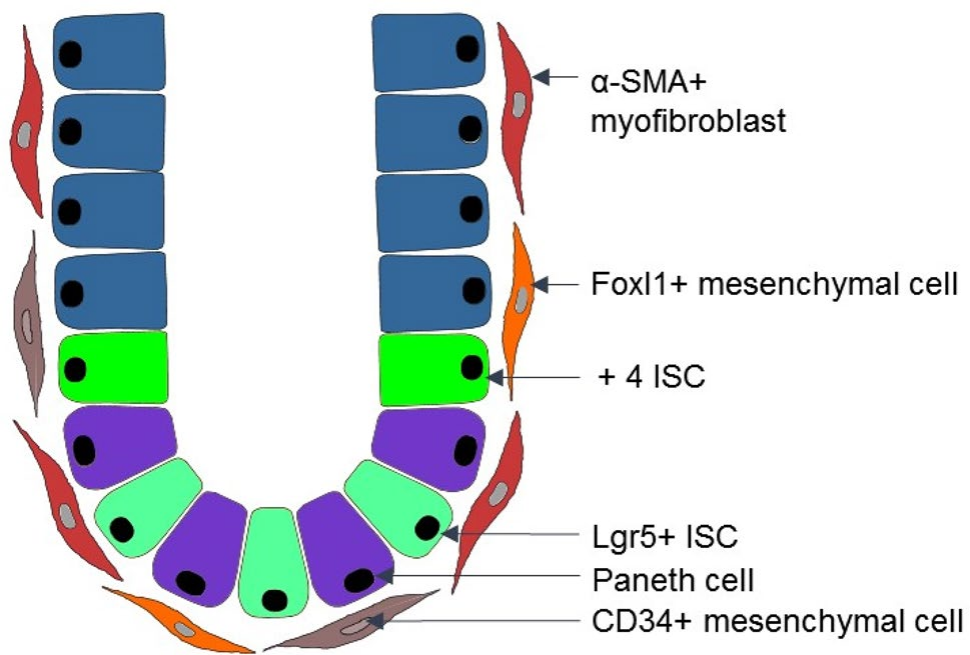
Scheme of the mesenchymal niche in the intestine. In the intestinal crypt, there are at least two subpopulations of intestinal stem cells (ISCs): Lgr5^+^ ISCs and + 4 ISCs that are responsible for the high regeneration capacity of the intestinal epithelium. Crypt cells, including ISCs, are in close contact with different types of mesenchymal cells such as: CD34^+^ mesenchymal cells, Foxl1^+^ mesenchymal cells and α-SMA^+^ myofibroblasts

Presence of IMFs during early intestinal organogenesis and their subepithelial localization in the adult intestine suggests that these mesenchymal cells may provide some crucial niche factors for the ISCs, and regulate proliferation and differentiation in the intestinal epithelium. Indeed, in situ hybridization revealed that subepithelial myofibroblasts can express Wnt ligands such as Wnt2b, Wnt4 and Wnt5b (Gregorieff et al. 2005), which strongly suggests that this stromal cell type can regulate Wnt signaling in the adjacent epithelial cells. Wnt pathway provides essential signals for ISCs and deregulations in this pathway are associated with the development of the intestinal cancer (Reya and Clevers 2005). However, surprisingly, the study of San Roman et al. has shown that deletion of porcupine (an enzyme responsible for posttranslational modifications and Wnt secretion) in Myh11^+^ cells (that include subepithelial myofibroblasts) has no phenotype in the intestinal crypt (San Roman et al. 2014) suggesting that there can be other niche cells compensating for the loss of Wnt secretion in subepithelial myofibroblasts. A possible explanation is, e.g., the presence of CD34^+^ mesenchymal cells (Stzepourginski et al. 2017) and Foxl1^+^ mesenchymal cells (Aoki et al. 2016) (Fig. 2). These cell types could provide compensatory signals, including Wnt ligands, for the ISCs in the absence of functional Wnts in the Myh11^+^ cells. Of note, CD34^+^ mesenchymal cells described by Stzepourginski et al. (2017) were studied only in ileum and colon. Moreover, Gli1^+^ subepithelial mesenchymal cells were proposed to be a source of Wnt ligands in the intestinal stem cell niche (Valenta et al. 2016); however, this stromal cell subpopulation remains uncharacterized. It is worth to add that besides mesenchymal cells, Paneth cells also can be a source of Wnt ligands for the ISCs, e.g., Wnt3 (Sato et al. 2011). Interestingly, depletion of Paneth cells has no phenotype in the intestinal crypt under homeostatic conditions (Durand et al. 2012). Altogether, this can suggest a cooperative work of epithelial (such as Paneth cells) and stromal cells in the intestinal stem cell niche. Existence of redundant mechanisms to maintain ISCs could protect against the loss of ISCs, which are necessary to maintain the pool of enterocytes whose primary function is nutrient absorption.

Although, for many years it has been believed that Wnts are critical regulators of the epithelial self-renewal in the intestinal crypt, data from in vitro (Glinka et al. 2011) and in vivo studies (Yan et al. 2017) revealed that in addition to Wnt ligands, R-Spondins also play a critical role in Wnt pathway. R-Spondins are secreted proteins that are involved in maintenance of the surface localization of a receptor-bound Wnt through regulation of the transmembrane E3 ligases Rnf43/Znrf3, which ultimately results in amplification of the Wnt signal (Farin et al. 2016). Yan et al. (2017) proposed that not Wnts, but rather R-Spondins may play a dominant role in self-renewal of Lgr5^+^ intestinal stem cells. Interestingly, R-Spondins are likely produced by stromal cells (Sigal et al. 2017); nonetheless, this requires detailed investigation in the future.

Besides Wnt ligands and R-Spondins, many other niche signals were shown to regulate intestinal epithelial cells. Here, among the molecules involved in the intestinal (myo)fibroblast—intestinal epithelial cell cross-talk are, e.g., hepatocyte growth factor (HGF) (Goke et al. 1998), prostaglandin E2 (PGE_2_) (Roulis et al. 2014), and periostin (Kikuchi et al. 2008). Moreover, IMFs, together with smooth muscle cells, were shown to guide intestinal epithelial regeneration in a dextran sulfate sodium (DSS) injury model via mechanism that involves microRNA-143/145 and insulin-like growth factor binding protein 5 (IGFBP5) (Chivukula et al. 2014). Furthermore, very recently angiopoietin-like protein 2, that is expressed in subepithelial myofibroblasts in colon, was demonstrated to play an important role during regeneration of the intestinal epithelium in two mouse models of intestinal injury (Horiguchi et al. 2017). Altogether, this suggests that IMFs regulate intestinal epithelial cells via various molecular mechanisms.

### IMFs in Disease

Increased number of α-SMA^+^ myofibroblasts was observed during both intestinal inflammation and intestinal tumor (Andoh et al. 2002; Powell et al. 2005). The role of IMFs during disease was previously thoroughly reviewed, e.g., by Powell et al. (2011), Roulis and Flavell (2016) and Koliaraki et al. (2017). Importantly, during intestinal inflammation and cancer, not only the number of IMFs is altered, but also changes in gene expression profiling and proteome profiling were detected in IMFs. For example, increased expression of inflammatory mediators such as interleukin 6, osteopontin, CXCL2 and CCL20 was found in carcinoma-associated fibroblasts (CAFs) derived from azoxymethane/dextran sodium sulfate (AOM/DSS) mice, an in vivo model of colitis-associated cancer, when compared to normal myofibroblasts (Torres et al. 2013). In addition, using the same research model, it was shown that tumor progression locus 2, a kinase that is expressed in IMFs, protects against colitis-associated cancer by regulating production of HGF (Koliaraki et al. 2012). Similarly, epimorphin, a mesenchymal protein, was shown to exhibit a protective role against colitis-associated cancer in AOM/DSS mouse model (Shaker et al. 2010). The potential limitations of the studies above are that: (1) AOM/DSS mouse model might not recapitulate the genetic landscape of human colitis-associated colorectal cancer, and (2) the differences between mouse and human immune system. A potential solution here is the application of human-derived organoid models and mouse models with humanized immune system to understand better the epithelial–stroma interactions in colitis-associated cancer.

Immunohistochemistry and gene expression data provided evidence that myofibroblasts could serve as a prognostic factor in colorectal cancer (Isella et al. 2015; Tsujino et al. 2007). It is worth to mention that in case of the global gene expression analyses of colorectal tumor tissue, myofibroblasts can be a source of “pseudo-EMT signals” (Calon et al. 2015), which should be taken into consideration when analyzing any gene expression data obtained from the whole tumor tissue. Additionally, stromal microRNA-21 was shown to have prognostic value in colorectal cancer (Nielsen et al. 2011). Excitingly, such stromal microRNA-21 can be associated with exosomes (Bhome et al. 2017), a type of extracellular vesicles that are produced by mammalian cells for the intercellular communication. Interestingly, CAF-derived exosomal microRNA-21 was shown to have an impact on colorectal cancer cell proliferation, resistance to chemotherapy and formation of liver metastases (Bhome et al. 2017). Besides that, mechanistically, myofibroblasts isolated from colon cancer tissue were shown to promote tumor cell invasion via mechanism that involves tenascin-C, scatter factor/HGF, RhoA and Rac (De Wever et al. 2004). Moreover, a study of Vermeulen et al. (2010) suggests that myofibroblasts could contribute to the “β-catenin paradox” (mosaic pattern of β*-*catenin nuclear localization*)* observed in colorectal cancer cells.

Inflammatory bowel disease (IBD) is characterized by epithelial injury and intestinal inflammation. IBD is a group of diseases that include ulcerative colitis and Crohn’s disease. One of the key cytokines that is involved in the pathogenesis of IBD is IL-33, which belongs to the IL-1 superfamily of cytokines; IL-33 is responsible for immune cell infiltration and Th2 responses (Miller 2011; Neurath 2014). Interestingly, the study of Sponheim et al. (2010) suggests that pericryptal myofibroblasts are a source of IL-33 in patients with ulcerative colitis, which highlights an important role of this cell type in the pathogenesis of ulcerative colitis and warrants for further studies on the role of pericryptal myofibroblasts in IBD. Moreover, the study of Messina et al. (2017) suggests that colonic CD146^+^ cells, that were shown to have features of IMFs (Signore et al. 2012), exhibit increased expression of HLA-DR, a major histocompatibility complex class II antigen. However, this requires more investigation. The findings should be confirmed using larger number of samples and functional studies should be performed. Additionally, it was shown that human IBD IMFs exhibit differential expression of distinct transforming growth factor β isoforms (McKaig et al. 2002). To summarize, IMFs are an important component of the stromal niche during IBD and intestinal cancer. Better understanding of the role of IMFs during pathogenesis of IBD and intestinal tumor can potentially lead to identification of new therapeutic targets for those diseases.

## Summary and Future Directions

To summarize, emerging data highlight the importance of mesenchymal–epithelial cross-talk in the intestine during homeostasis, regeneration after an injury and chronic diseases. Here, we particularly focused on subepithelial myofibroblasts that surround the intestinal crypt. Many studies pointed out the important role of the subepithelial myofibroblasts in regulation of intestinal epithelial proliferation via different molecular mechanisms that involve, e.g., HGF, PGE_2_, periostin, microRNA-143/145 and IGFBP5. Still, many questions remain to be answered. For example, it would be interesting to decipher whether subepithelial myofibroblasts can activate quiescent ISCs and if migration of crypt cells along the crypt–villus is regulated autonomously or rather by subepithelial myofibroblasts?. In a mammalian organism, there are multiple mechanisms responsible for the maintenance of adult stem cells. One example is asymmetric organelle segregation during cell division (Ouellet and Barral 2012). The study of Katajisto et al. (2015) demonstrated that young mitochondria are preferentially distributed to stemlike cells during mitosis of mammary epithelial cells. It would be exciting to unpuzzle whether subepithelial myofibroblasts could regulate segregation of mitochondria in the neighboring ISCs. Given the stromal cell heterogeneity in the intestinal stem cell niche, it would be also interesting to study the relationship of subepithelial myofibroblasts with other types of mesenchymal cells such as CD34^+^ mesenchymal cells and Foxl1^+^ mesenchymal cells. Moreover, differentiation status of CD34^+^ and Foxl1^+^ mesenchymal cells remains unclear: can these cell types act as progenitor cells for the myofibroblast syncytium?. It is also unknown whether CD34^+^ mesenchymal cells are the same cells as Foxl1^+^ mesenchymal cells.

Aberrant niche signaling was detected in various human diseases, including colorectal cancer and IBD. IMFs were identified as one of key components of the stromal niche in both colorectal cancer and IBD, where IMFs were suggested, e.g., to be a source of inflammatory mediators. Future studies should provide more input into the precise role of subepithelial myofibroblasts in the regulation of immune response in IBD. It would be also interesting to study whether subepithelial myofibroblasts can provide signals promoting self-renewal of colon cancer stem cells. And, as niche factors are especially important during epithelial homeostasis and very early stages of intestinal tumor growth (Fujii et al. 2016), it would also be intriguing to investigate whether myofibroblast-derived niche factors can promote tumor initiation process in the intestinal epithelium. Overall, increasing the knowledge on the myofibroblasts–intestinal epithelium cross-talk in the intestinal stem cell niche during homeostasis and disease can lead to identification of novel therapeutic targets, e.g., for colon cancer and IBD.

**Fig. 3:**
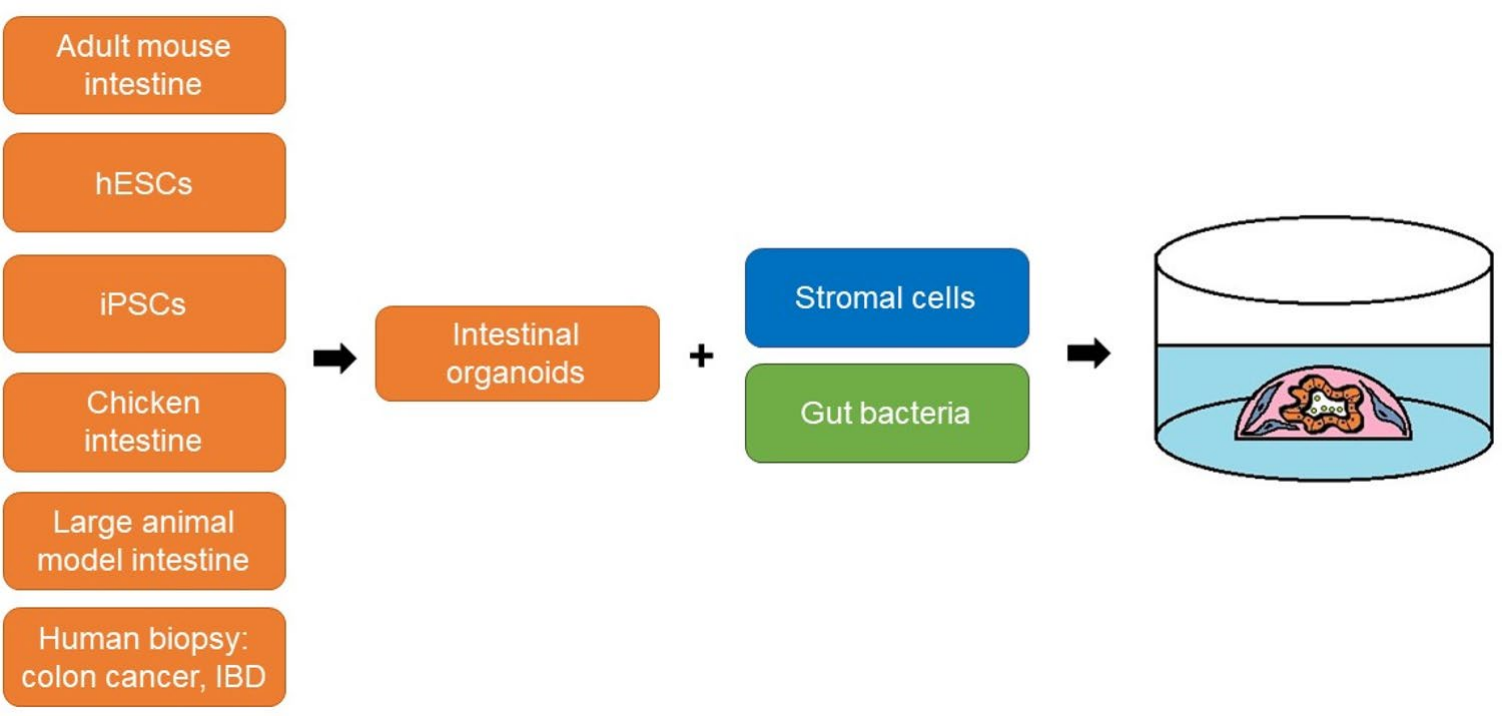
Modifications of the mini-gut culture system to reconstruct the intestinal tissue microenvironment in vitro. For the co-culture, intestinal organoids can be combined with stromal cells or/and live bacteria. A source of primary intestinal epithelial cells can be, e.g., adult mouse intestinal tissue, chicken intestinal tissue, human embryonic stem cells (hESCs), induced pluripotent stem cells (iPSCs), and biopsy samples from patients with colon cancer or inflammatory bowel disease (IBD)

Recent advances in 3D cell biology have enabled the reconstruction of the intestinal stem cell niche in vitro. Since 2009, it has been possible to maintain ISC in vitro in a long-term culture system known as crypt culture or mini-gut culture (Pastula and Quante 2014; Sato et al. 2009). Recently, such a mini-gut culture has been further improved by incorporating the stromal microenvironment such as IMFs or neurons (Lahar et al. 2011; Lei et al. 2014; Pastula et al. 2014, 2016a, 2016b) (Fig. 3). For the stromal niche modeling in vitro, mesenchymal cells can be either mixed together with epithelial cells and Matrigel (Pastula et al. 2016b) or epithelial organoids can be seeded on the mesenchymal cell monolayer (Holmberg et al. 2017; Lahar et al. 2011; Lei et al. 2014). In addition, IMFs and epithelial organoids can be seeded in separate layers in a Transwell (Pastula et al. 2016b). Additionally, advances in 3D cell culture systems led to development of intestinal organoid cultures derived from human embryonic stem cells and human-induced pluripotent stem cells (Crespo et al. 2017; Rodansky et al. 2015), as well as intestinal organoids derived from large animal models (Khalil et al. 2016). Intriguingly, not only stromal cells, but also live bacteria such as *Lactobacillus acidophilus* (a part of the normal bacterial flora in our organism) can be added to the intestinal organoid cultures (Pierzchalska et al. 2017) (Fig. 3), that provides an additional level of complexity to the epithelial intestinal organoids, and offers a valuable tool to study interactions between the gut microbiome and the intestinal epithelium. Since it is possible to culture organoids derived from biopsy samples from patients with colon cancer (van de Wetering et al. 2015) and IBD (Dotti et al. 2017), such human-derived organoids could be used for the co-cultures with different types of intestinal mesenchymal cells, immune cells and microbiota, to better mimic organs for disease modeling in vitro. Recently, a biobank of human-derived organoids derived from multiple organs and also diseased tissue, including colon cancer and IBD, was established (Dutta et al. 2017). In addition, it would very useful to set up a living biobank of different types of intestinal mesenchymal cells and gut microbiota derived from patients suffering from colon cancer and IBD.

Certainly, application of in vitro 3D organ models, such as those described above, for further studies on the role of microenvironment–epithelial interactions in the intestinal stem cell niche will lead in the future to new exciting discoveries in both basic and translational research.
